# A Comparative Study of Spinal Deformity and Plantar Pressure according to the Static Standing Posture of Female Adolescents with or without Idiopathic Scoliosis

**Published:** 2019-02

**Authors:** Kewwan KIM, David R. MULLINEAUX, Kyoungkyu JEON

**Affiliations:** 1. Division of Exercise and Health Science, College of Arts and Physical Education, Incheon National University, Incheon, Korea; 2. School of Sport and Exercise Science, College of Social Sciences, University of Lincoln, Lincoln, United Kingdom; 3. Sport Science Institute, Incheon National University, Incheon, Republic of Korea

## Dear Editor-in-Chief

Scoliosis can be defined as complex spinal deformities, including thoracic kyphosis and spinal rotation, where the rotation of the entire spine causes a lateral curvature and asymmetry (1, 2). Scoliosis is associated with a malalignment due to wrong posture and rapid growth during adolescence, between 11 and 17 yr of age (3). Neglecting such postural abnormalities and imbalance due to asymmetry can negatively impact healthy growth, and it is highly correlated with other pathological diseases such as chronic back pain (4). Therefore, basic approaches continued monitoring, and postural management must be actively implemented to prevent and treat idiopathic scoliosis in adolescents in their prime growing years, in consideration of age and the severity of the disease (1). This study aimed to develop a method to use the findings as basic data for improving abnormal curvature and asymmetry and contribute to the promotion of public health efforts at improving spinal health in daily life.

Overall, 134 female primary school students participated in the “Scoliosis Early Screening and Prevention Program” conducted by Incheon National University (INU) in 2015. The study population included 74 adolescents with idiopathic scoliosis (age 11.24±2.01 years, height 147.84±9.84 cm, weight 40.86±10.21 kg) and 60 healthy adolescents (age 11.43±.93 yr, height 146.30±9.33 cm, weight 38.13±7.87 kg).

The study was conducted after receiving the necessary consent from the students and the parents. All participants provided written informed consent, and the Institutional Review Boards at approved the study INU.

A 3-dimensional spine structure analysis and plantar pressure device (Formetric 4D and Pedoscan, DIERS International GmbH, Schlangenbad, Germany) was used to measure and compare the factors that affect spinal deformity and the rotation of center of pressure in the static standing posture. These instruments allowed accurate and rapid measurements to be obtained since they were performed by surface tomography using a rasterized video method on four anatomical landmarks (cervical 7, the left and right posterior superior iliac spine, and the center of the sacral) on the back of the body. The device provided data that were objective and highly reproducible without the risk of radiation exposure (5). We used these data for comparisons with plain radiographs, and the device could also be used to diagnose spinal joint asymmetry and scoliosis (6). The means and standard deviations of all the measured data were derived using SPSS 23.0 (IBM Corp, Armonk, NY, USA). An independent samples t-test was used for comparisons between the groups, and the statistical significance level was set to α=.05 (Table 1).

**Table 1:** Results of three-dimensional spinal structural and plantar pressure analysis

**Variables**		**Scoliosis Group (n=74)**	**Normal Group (n=60)**
Scoliosis Angle (deg)		18.62±3.86	6.83±1.77^***^
Kyphotic Angle (deg)		47.69±8.74	42.15±9.22^**^
Lordotic Angle (deg)		37.83±7.15	37.63±9.20
Pelvic	Obliquity (deg)	0.14±2.32	0.32±2.56
	Inclination Symmetry (deg)	19.15±4.51	21.67±6.13^**^
	Torsion (deg)	1.05±1.76	1.42±2.21
Maximum	Left (%)	14.53±5.97	13.89±5.98
Pressure	Right (%)	11.93±3.77	10.81±4.42
Center of Pressure (COP) Rotation (deg)		4.34±2.73	3.36±2.72^*^

Values are Mean±SD,

* *P*<0.05,

** *P*<0.01,

*** *P*<0.001

The adolescents with idiopathic scoliosis showed statistical significance in scoliosis angle, kyphotic angle, and asymmetric angle of pelvic inclination compared to the healthy adolescents. The form of idiopathic scoliosis in adolescents is closely associated with deformities such as thoracic kyphosis and pelvic asymmetry. Moreover, significance was found in the rotational angle of the center of plantar pressure, indicating that spinal deformity influenced the static standing posture. Based on our findings, regular scheduled, systematic, early screening is needed to maintain proper posture in adolescents in their growing stages are also needed for public health.
